# Acute Transverse Myelitis as an Unusual Complication of Dengue Fever: A Case Report and Literature Review

**DOI:** 10.7759/cureus.54074

**Published:** 2024-02-12

**Authors:** FNU Karishma, FNU Harsha, Sindhu Rani, Saman Siddique, Kiran Kumari, FNU Mainka, Hira Nasir

**Affiliations:** 1 Internal Medicine, Ghulam Muhammad Mahar Medical College, Sukkur, PAK; 2 Medicine, Hamdard University, Karachi, PAK; 3 Internal Medicine, Peoples University of Medical and Health Sciences, Karachi, PAK; 4 Medicine and Surgery, Shaheed Mohtarma Benazir Bhutto Medical University, Karachi, PAK; 5 Internal Medicine, Mayo Hospital, Lahore, PAK

**Keywords:** dengue ns1, post dengue transverse myelitis, acute transverse myelitis (atm), complications of dengue fever, dengue virus infection

## Abstract

Dengue fever, the most prevalent arbovirus disease, has a broad spectrum of clinical manifestations, ranging from asymptomatic to dengue hemorrhagic fever and dengue shock syndrome. Dengue fever has the potential to involve the nervous system. Acute transverse myelitis is a life-threatening complication of dengue fever, though rarely reported. We report a case of dengue fever-induced transverse myelitis in a 51-year-old male who presented with progressive paraplegia, sensory disturbance, and urinary retention preceded by a febrile illness, vomiting, and retro-orbital pain two weeks before. His serology was positive for immunoglobulin M (IgM) to dengue virus and non-structural protein (NS-1). Magnetic resonance imaging revealed hyperintense signals suggestive of acute transverse myelitis. After ruling out all other possible causes, a possible diagnosis of dengue fever-induced transverse myelitis was made. His condition improved gradually after starting methylprednisolone.

## Introduction

Dengue fever, a viral illness transmitted by the Aedes mosquito, is prevalent in tropical and subtropical regions, notably in the American and Asian populations. Dengue fever has a broad spectrum of clinical manifestations and clinical syndromes ranging from sub-clinical infections to life-threatening infections such as dengue shock syndrome and dengue hemorrhagic fever [1]. Patients with dengue fever generally present with fever, nausea, vomiting, and abdominal and retro-orbital pain [2]. Dengue fever has the potential to involve the multi-organ system and has also been associated with cardiomyopathy, hepatic injury, depression, pneumonia, iritis, and orchitis. Neurological complications of dengue fever have also been reported [3]. Transverse myelitis is a life-threatening complication of dengue fever, though rarely reported, and only a limited number of cases have been published [4]. We report a case of acute transverse myelitis induced by dengue fever.

## Case presentation

A 51-year-old male was brought to the emergency department with walking difficulty, sensory disturbance, and urinary retention. He first developed lower extremity weakness three days earlier, followed by sensory disturbance and urinary retention. These symptoms had gradual onset, progressive, with no aggravating and relieving factors. He also reported high-grade fever, myalgia, retro-orbital pain, and multiple episodes of vomiting two weeks ago. His blood picture showed decreased platelet count on serial complete blood counts, dengue serology was positive for nonstructural protein 1 (NS1), and he was diagnosed with dengue fever. He had no history of abdominal pain, sore throat, cough, ear discharge, skin rash, backache, or trauma.

On examination, he was hemodynamically stable and well-oriented to time, place, and person. On neurological examination, he had decreased motor strength in his lower extremities with a power of grade 1/5 on the left side and grade 0/5 on the left side, with normal strength in his upper extremities. He had hypoesthesia with absent reflexes in his lower limbs, and the sensory level was noted at the T-6 dermatome with impaired joint position sensation of the lower limbs. He had grade B paresis on the Frankel Grade.

**Figure 1:**
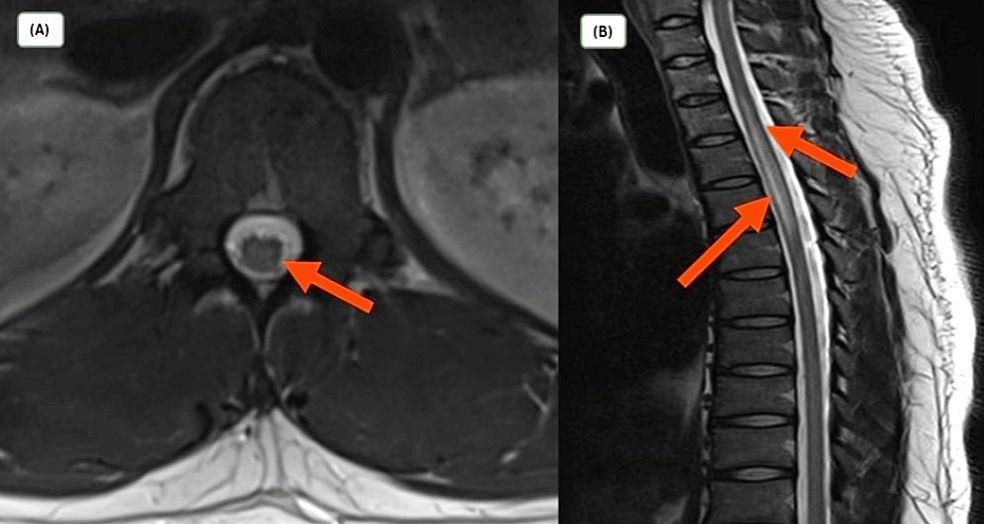
T-2 weighted spinal MRI demonstrating hyperintense signals from the T2-T6 vertebrae (A, B)

Higher mental function and cranial nerve examinations were within normal limits. Cardiovascular and respiratory system examinations were unremarkable. He underwent brain magnetic resonance imaging (MRI), which was normal. Results of cerebrospinal fluid (CSF) analysis are shown in Table 1. Spine T-2 weighted MRI revealed hyperintense signals in the thoracic T2-T6 region, and nerve conduction study findings were suggestive of transverse myelitis (Figure 1).

**Table 1:** Results of the CSF analysis CSF: cerebrospinal fluid analysis, VDRL: venereal disease research laboratory

CSF	Lab Values	Reference Range
Color	Colorless	Colorless
Opening pressure	12	06-20 cmH_2_O
Proteins	71	14-47 mg/dl
Glucose	87	42-75 mg/dl
Red cell count	11	0-1/mm^3^
White cell count	19	0-6/mm^3^
VDRL	Nonreactive	Nonreactive
Gram stain	Negative	Negative
Bacterial antigen	Negative	Negative

Repeated serology for dengue fever was positive for NS-1 and immunoglobulin (Ig) M and IgG. Further workup revealed normal blood cell count and serum biochemistry. His serology was negative for coronavirus disease 2019, syphilis, human immunodeficiency virus (HIV), herpes virus serology, viral hepatitis, and autoimmune workup (Table 2).

**Table 2:** Detailed workup to rule out other etiologies of transverse myelitis COVID-19: coronavirus disease 2019, HCV: hepatitis C virus, HIV: human immunodeficiency virus, VDRL: venereal disease research laboratory, PCR: polymerase chain reaction, HSV: herpes simplex virus

Workup	Results
COVID-19 PCR	Negative
Hepatitis B surface antigen	Negative
Anti HCV antibody	Negative
Anti-nuclear antibody test	1:40
Rheumatoid factor	<5 IU/ml
HIV antigen	Negative
VDRL	Negative
HSV PCR	Negative

He was diagnosed with acute transverse myelitis induced by dengue fever after ruling out all other possible etiologies. His condition improved gradually after starting high-dose intravenous methylprednisolone (1g/daily) for five days, followed by tapering oral steroids and physiotherapy. He was discharged 10 days later on tapering oral steroids. On his recent visit four weeks later, he was doing well, with improved symptoms.

## Discussion

**Figure 2:**
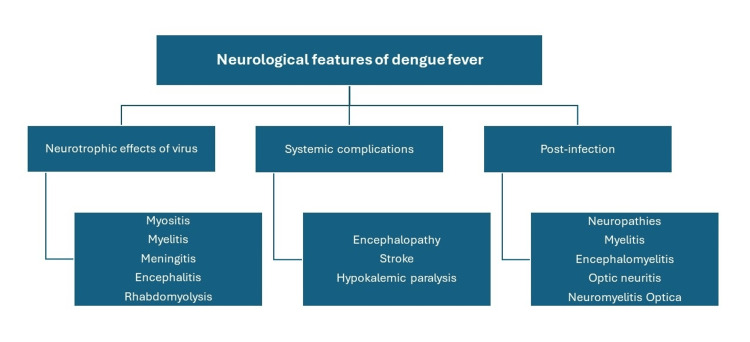
Neurological features of dengue fever Image credit: Saman Siddique, Hira Nasir

**Figure 3:**
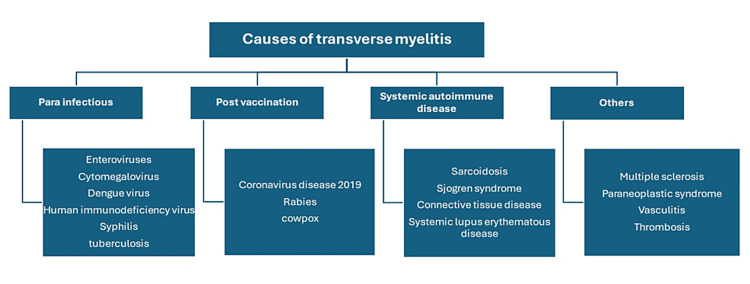
Etiology of transverse myelitis Image credit: Kiran Kumari, FNU Harsha

Dengue fever has the potential to involve the nervous system (Figure 2) [3]. Transverse myelitis is an uncommon, life-threatening, inflammatory neurological disorder characterized by rapid onset of weakness, sensory disturbance, and bladder or bowel dysfunction. It has a wide spectrum of etiologies such as para-infectious, post-vaccination, systemic autoimmune diseases, and vasculitis (Figure 3) [5,6]. Transverse myelitis induced by dengue fever is uncommon, and only a limited number of cases have been published. A systemic review of neurological manifestations of dengue fever revealed that the incidence of transverse myelitis is low, and out of 2672 cases, only 61 patients were diagnosed with transverse myelitis after dengue fever. Of those, 32 patients were male, and the mean age of presentation was 33.1 years, with an average time of 11.7 days from dengue to the first onset of neurological symptoms of transverse myelitis [4]. We have tabulated the reported cases of transverse myelitis complicated by dengue fever reported after 2018 (Table 3) [3,7-11].

**Table 3:** Reported cases of transverse myelitis induced by dengue fever M: male, F: female, IgM: Immunoglobulin M, IgG: Immunoglobulin G, T2-W: T2-weighted, T: thoracic. NS-1: non-structural protein-1 Source: [3,7-11]

Author et al.	Age (year)/sex	Dengue diagnosis	Days to neurological symptoms	Symptoms	Imaging	Management	Outcome
Chaudhry et al. [7]	55/F	NS-1, IgM, IgG	4	Urinary retention, paraplegia, sensory loss	Hyperintense signals on T2-W at T9-T10	Steroids, physiotherapy	Recovered
Farias et al. [8]	53/M	IgM	3	Lower limb weakness, urinary retention	Hyperintense signals on T2 at the T4/5 level	High-dose steroids	Recovered
Malik et al. [3]	15/M	NS-1	42	Lumber pain, bowel, and bladder dysfunction	T2 hyperintense signals from T5 to conus medullaris	High-dose steroids, physiotherapy	Recovered
Landais et al. [9]	24/F	NS-1, IgM, IgG	7	Paraparesis, urinary retention	T2 hyperintense lesions at the cervical and thoracic level	High-dose steroids	Sphincter disorder persisted
Lana-Piexoto et al. [10]	41/M	IgM	24	Lower limb weakness, sensory impairment	T2 hyperintense signal intensity from T5 to the conus medullaris	High-dose steroids	Recovered
Tan et al. [11]	61/F	NS-1	6	Lower limb weakness, urinary retention	Hyperintensity signals on T2 at T9-T10	High-dose steroids, physiotherapy	Recovered

The pathophysiology of dengue fever-induced transverse myelitis is not yet defined. Possible mechanisms include the direct neurotropic effect of the virus on the nervous system or post-infectious complications [10,11]. A direct neurotropic effect has been reported in a few cases of myelitis, with the isolation of viral antigens in the CSF of the patients in an early phase of infection. Viral antigens can be found in the spinal cord, brain, and brainstem in humans, infecting the neural tissues [12]. Dengue virus has also been reported to replicate within CSF passing through the blood-brain barrier disrupted by intense inflammatory response. Transverse myelitis associated with post-infectious dengue virus involves a transient stimulated autoimmune response through molecular mimicry and non-specific activation of auto-reactivated T-cell clones, directing myelin or other nervous tissues and stimulating a cascade of inflammatory response [13]. Post-infectious myelitis typically manifests one or two weeks after the onset of initial dengue fever manifestations, whereas para-infectious dengue myelitis can occur within the first week of infection [12,13].

Spinal MRI is the modality of choice in diagnosing transverse myelitis, and the presence of T-2 weighted hyperintense signals is suggestive of myelitis [14]. Detecting dengue virus antigen in CSF serves as an indicator of acute phase infection caused by dengue virus. Notably, the window for detecting the virus in serum and CSF is brief; however, the test is highly efficacious during the viremia phase. Hence, molecular testing is preferred within the initial week following the symptoms, and beyond this period, serologic testing becomes the preferred diagnostic method for dengue infection [15]. Ruling out other viral causes is also recommended. Dengue fever is managed conservatively depending on the severity, as there is no recommended viral treatment. Dengue fever-induced transverse myelitis is managed with high-dose steroids [14,15]. Our patient was diagnosed with dengue-induced myelitis, and his condition improved after starting steroids.

## Conclusions

Although rare, transverse myelitis is a life-threatening complication of dengue fever that clinicians should be aware of, particularly in endemic regions. Transverse myelitis has a favorable prognosis in case of early recognition and aggressive management with high-dose steroids. Our case highlights the need for careful observation in the monitoring of dengue patients for neurological complications, enabling timely management and improved outcomes.
